# Sex-dependent circadian alterations of both central and peripheral clock genes expression and gut–microbiota composition during activity-based anorexia in mice

**DOI:** 10.1186/s13293-023-00576-x

**Published:** 2024-01-12

**Authors:** Colin Salaün, Marine Courvalet, Léna Rousseau, Kévin Cailleux, Jonathan Breton, Christine Bôle-Feysot, Charlène Guérin, Marion Huré, Alexis Goichon, Jean-Claude do Rego, Pierre Déchelotte, David Ribet, Najate Achamrah, Moïse Coëffier

**Affiliations:** 1 Univ Rouen Normandie, INSERM, Normandie Univ, ADEN UMR 1073, Nutrition Inflammation and Microbiota Gut Brain Axis, UFR Santé, 22 Boulevard Gambetta, 76183 Rouen Cedex, France; 2 Univ Rouen Normandie, Institute for Research and Innovation in Biomedicine (IRIB), 76000 Rouen, France; 3 Univ Rouen Normandie, Inserm, CNRS, Normandie Univ, HERACLES US 51 UAR 2026, Behavioural Analysis Platform SCAC, 76000 Rouen, France; 4 Department of Nutrition, CHU Rouen, 76000 Rouen, France

## Abstract

**Rationale:**

Patients with anorexia nervosa (AN) often present sleep disorders and circadian hormonal dysregulation. The role of the microbiota–gut–brain axis in the regulation of feeding behavior has emerged during the last decades but its relationships with the circadian rhythm remains poorly documented. Thus, we aimed to characterize the circadian clock genes expression in peripheral and central tissues in the activity-based anorexia mouse model (ABA), as well as the dynamics of the gut–microbiota composition.

**Methods:**

From day 1 to day 17, male and female C57Bl/6 mice were submitted or not to the ABA protocol (ABA and control (CT) groups), which combines a progressive limited access to food and a free access to a running wheel. At day 17, fasted CT and ABA mice were euthanized after either resting (EoR) or activity (EoA) phase (*n* = 10–12 per group). Circadian clock genes expression was assessed by RT-qPCR on peripheral (liver, colon and ileum) and central (hypothalamic suprachiasmatic nucleus or SCN) tissues. Cecal bacterial *taxa* abundances were evaluated by qPCR. Data were compared by two-way ANOVA followed by post-tests.

**Results:**

ABA mice exhibited a lower food intake, a body weight loss and an increase of diurnal physical activity that differ according with the sex. Interestingly, in the SCN, only ABA female mice exhibited altered circadian clock genes expression (*Bmal1, Per1, Per2, Cry1, Cry2*). In the intestinal tract, modification of clock genes expression was also more marked in females compared to males. For instance, in the ileum, female mice showed alteration of *Bmal1*, *Clock*, *Per1*, *Per2*, *Cry1*, *Cry2* and *Rev-erbα* mRNA levels, while only *Per2* and *Cry1* mRNAs were affected by ABA model in males. By contrast, in the liver, clock genes expression was more markedly affected in males compared to females in response to ABA. Finally, circadian variations of gut–bacteria abundances were observed in both male and female mice and sex-dependent alteration were observed in response to the ABA model.

**Conclusions:**

This study shows that alteration of circadian clock genes expression at both peripheral and central levels occurs in response to the ABA model. In addition, our data underline that circadian variations of the gut–microbiota composition are sex-dependent.

**Supplementary Information:**

The online version contains supplementary material available at 10.1186/s13293-023-00576-x.

## Plain language summary

Anorexia nervosa is an eating disorder with a female predominance. However, the underlying pathophysiological mechanisms are still incompletely understood. Patients with anorexia nervosa often show alterations in circadian rhythm, including sleep disorders and modifications in hormone circadian rhythm. The circadian rhythm is controlled in the central nervous system, particularly in the suprachiasmatic nucleus, but clocks have also been described in peripheral tissues. To better understand the putative role of circadian rhythm in the pathophysiology of anorexia nervosa, we have conducted an experimental study in a rodent model of anorexia nervosa called “activity-based anorexia” on both males and females. Interestingly, we observed that the expression of genes involved in the circadian rhythm is affected by the activity-based anorexia model in both the suprachiasmatic nucleus and peripheral tissues, such as the small intestine and liver. In addition, gut–microbiota also shows circadian variation. Interestingly, the anorexia-induced alterations of circadian variations (clock genes expression and gut–microbiota composition) are sex- and tissue-dependent. For instance, female mice exhibited more marked alterations in the ileum, whereas, in males, modifications were more pronounced in the liver. This study highlights sex-dependent alterations of circadian clock genes expression and of gut–microbiota in response to the anorexia rodent model. Further experiments should be performed to investigate the contribution of these mechanisms in the etiology of anorexia nervosa and the higher prevalence in females.

**Supplementary Information:**

The online version contains supplementary material available at 10.1186/s13293-023-00576-x.

## Highlights

- In response to activity-based anorexia, a rodent model mimicking anorexia nervosa, the activity wheel is affected in a sex-dependent manner.
- In response to activity-based anorexia, circadian clock genes changes are sex- and tissue-dependent. In peripheral tissues, males and females showed more marked alterations of circadian clock genes expression, respectively, in the liver and in the ileum.
- Gut–microbiota composition is differently affected by activity-based anorexia in female and male mice.

**Supplementary Information:**

The online version contains supplementary material available at 10.1186/s13293-023-00576-x.

## Introduction

Anorexia nervosa (AN), an eating disorder defined by the fifth edition of the Diagnostic and Statistical Manual of Mental Disorders (DSM-5) [9], is characterized by a body mass index (BMI) lower than 18.5 kg/m^2^, an intense fear of gaining weight leading to a negative energy balance. AN is also frequently associated with compensatory mechanisms, such as physical hyperactivity in 30–80% of patients [14, 18] and an altered body shape perception. In addition, AN is the psychiatric disorder with the highest mortality rate, around 1–5% after 10 years [27]. The lifetime prevalence of AN is increasing with about 1.4% in women and 0.2% in men [22]. Nevertheless, studies in both females and males are needed because of differences in the pathophysiology, in the incidence peaks, in brain development and hormonal changes [48]. The etiology of AN is multifactorial and remains not well-understood [57]. During the last decades, alterations of metabolic pathways and the role of microbiota–gut–brain axis in AN have been highlighted. Indeed, a gut–microbiota dysbiosis has been reported in several studies both in AN patients [5, 6, 20] and in animal models [6, 7, 17, 51, 52]. In addition, sleep disorders [2, 3] and dysregulations of hormonal circadian rhythm [26] have been reported in AN patients. For instance, circadian plasma leptin levels are altered in AN patients and are associated with insulin and cortisol levels [26].

Circadian rhythm is regulated by a master clock in the suprachiasmatic nucleus (SCN) that is mainly controlled by light/dark cycle, while peripheral clocks are impacted by food intake. The primary actors involve CLOCK/BMAL1 (also named ARNTL) couple interaction regulated by PER/CRY couple. At the second level of regulation, NR1D1 (commonly named *REV-ERBΑ*) regulates *Bmal1* transcription. Clock genes expression differs between tissues [30, 55] as referenced in the database of circadian transcriptional profiles, CircaDB [39]. Interestingly, gut–microbiota also seems to affect peripheral clocks, particularly in the intestine [45, 47]. Germ-free mice exhibit changes in liver circadian genes expression, such as *Cry1*, *Per1* and *Per2* [34]. By contrast, disruption of circadian rhythm in mice induced modifications: of the intestinal barrier function, of bacteria recognition pathways and of gut–microbiota composition with decreased *Lactobacillus johnsonnii* abundance [16]. These data underline that circadian rhythm alterations might affect the microbiota–gut–brain axis and might contribute to AN pathophysiology.

The most relevant rodent model for AN is the activity-based anorexia (ABA) model [43, 44]. Developed in both rats and mice, it consists in a reduction of the duration to food access combined with a free access to a running wheel to mimic both the undernutrition and the hyperactivity observed in AN patients. ABA rodents showed a reduced food intake, a marked body weight loss [29, 37], a dysbiosis of the gut–microbiota [7, 52], reduced brain volumes [52], a decrease of leptin plasma level [49], as well as circadian rhythm alterations with a diurnal food anticipatory activity or FAA [1, 21]. In addition, a sex-dependent response to ABA was previously described. Males showed more marked responses to the ABA model with a lower food intake and a higher body weight loss than females, associated with sex-dependent alterations of physical activity and food behavior regulatory neuropeptides levels [1, 50]. In addition, the response to drugs or interventions also appears sex-dependents, such as the response to ketamine injections [24], to naloxone treatment [13] or to gut–microbiota depletion [49].

Thus, we aimed to characterize the circadian transcriptional profile at the central and peripheral levels in both female and male mice submitted to the ABA model. In addition, we also evaluated the circadian changes in gut–microbiota composition in this animal model of anorexia.

## Materials and methods

### Experimental procedure

All experimental procedures were approved by an ethical committee (APAFIS#9473-2016072016008305 v6). After 6 days of acclimatization to inversed cycle (dark phase between 10:00 AM and 10:00 PM) and a standard diet (SAFE A03 SP-10, Augy, France) in the SCAC animal behavioural platform, 48 male and 48 female 9-week-old C57BL/6 mice (Janvier Labs, Le Genest-Saint-Isle, France) were randomly assigned to either the control (CT) or activity-based anorexia (ABA) groups. CT mice were placed in standard cages and ABA mice were housed in cages equipped with a monitored running wheel (ActiviWheel software Intellibio, Seichamps, France). CT mice had free access to food and water throughout the study. After 5 days of acclimatization with free access to food and water, ABA mice had a limited access to food from 6 h/day at day 6 to 3 h/day at day 9 and until the end of experiment at day 17. Food access was provided at the beginning of the dark phase (10:00 AM). Food intake and body weight were registered daily at the end of the light phase (Additional file 1: Fig. S1). For ethical reasons, two males ABA mice were excluded from the protocol.

To investigate a putative shift in circadian rhythm, mice were euthanized at two timepoints: at the end of the activity phase (EoA), between 9:00 PM and 11:00 PM, or at the end of the resting phase (EoR), between 9:00 AM and 11:00 AM. Thus, there were four groups for both females and males: CT–EoA/CT–EoR/ABA–EoA/ABA–EoR with *n* = 11–12 per group (Additional file 2: Table S1). To avoid interaction of food intake on circadian clock genes expression, pellets were removed from both CT and ABA mice at 1:00 PM. After 9 or 21 h of fasting for EoA or EoR groups, respectively, mice were anesthetized with an intraperitoneal injection of Ketamine (Boehringer Ingelheim, COVETO, Caen, France; 100 mg/kg) and Xylazine (Bayer HealthCare, Puteaux, France; 10 mg/kg) diluted in NaCl 0.9%. Then, blood was collected on heparinized vacutainer tubes in the abdominal aorta and tissues were sampled as described in the Additional file 1 (Fig. S1). Blood samples were then centrifuged at 3000×*g* for 15 min at 4 °C and plasma was collected. All samples (plasma, hypothalamus containing SCN, liver, colon, ileum, and cecal content) were immediately frozen in liquid nitrogen and stored at − 80 °C until analysis (see Additional file 1: Fig. S1 for details).

**Table 1:** Primers used to study circadian rhythm by RT-qPCR

Target gene	Sense	Oligonucleotides	Tm uses (°C)
*Gapdh*	Forward	CATCACTGC CACCCAGAAGA	60.0
	Reverse	AAGTCG CAG GAG ACA ACC T	
	Reverse	TTCCTCAACACCACATGAGC	
*Bmal1*	Forward	CACCGTGCTAAGGATGGCTG	55.4
	Reverse	CTGCTGCCCTGAGAATTAGG	
*Clock*	Forward	GGACACGGATGATAGAGGCA	56.3
	Reverse	GTATCATGTGCTGGCCTGTG	
*Rev-erbα*	Forward	CCCTGGACTCCAATAACAACACA	61.0
	Reverse	GCCATTGGAGCTGTCACTGTAG	
*Per1*	Forward	CCAGATTGGTGGAGGTTACTGAGT	61.0
	Reverse	GCGAGAGTCTTCTTGGAGCAGTAG	
*Per2*	Forward	TTCCACTATGTGACAGCGGAGG	61.0
	Reverse	CGTATCCATTCATGTCGGGCTC	
*Cry1*	Forward	GGAAGGAACGAGATGCAGCT	59.4
	Reverse	AGTGGCTCCATCTTGCTGAC	
*Cry2*	Forward	GGGACTCTGTCTATTGGCATCTG	57.1
	Reverse	GTCACTCTAGCCCGCTTGGT	
	Reverse	AGAGGTGGTGTAAGCCATGC	

### RT-qPCR

TRIzol-chloroform (Invitrogen, Carlsbad, CA, USA and Merck, Darmstadt, Germany, respectively) RNA extraction was performed as previously described [10, 12] on SCN, liver, colon and ileum. After DNAse treatment (Promega, Charbonnières-les-Bains, France), reverse transcription of RNAs into cDNAs was performed with M-MLV (Invitrogen, Carlsbad, US). The circadian genes studied were *Rev-erbα*, *Bmal1*, *Clock*, *Per1* and *2*, *Cry 1* and *2* and housekeeping gene was *Gapdh*. Primers used for circadian rhythm are displayed in Table 1. All qPCRs used SYBR Green technology (Bio-Rad Laboratories, Marnes la Coquette, France) and were performed on an Eppendorf RealPlex Mastercycler (Eppendorf, Hambourg, Germany) or on a Bio-Rad CFX96 manager (Bio-Rad Laboratories, Marnes la Coquette, France). The values were obtained by the conversion of cycle threshold on concentration value using a standard curve. mRNA levels of genes of interest were normalized by mRNA levels of *Gapdh*.

### Gut–microbiota analyses

DNA was extracted from cecal content and qPCR were performed as previously described [7], using QIAmp DNA stool kit (Qiagen, Hilden, Germany). Then, qPCRs were performed on Eppendorf RealPlex Mastercycler or on a Bio-Rad CFX96 manager to determine the abundance of different bacterial taxa. The taxa-specific primers used for gut–microbiota quantification are displayed in Table 2.

**Table 2:** Primers used for gut–microbiota abundance on cecal DNA with qPCR

Target *Taxa*	Sense	Oligonucleotides	Tm uses (°C)
*Eubacteria*	Forward	ACTCCTACGGGAGGCAGCAG	60
	Reverse	ATTACCGCGGCTGCTGG	
*Bacteroidetes* (newly named *Bacteroidota*)	Forward	CATGTGGTTTAATTCGATGAT	51
	Reverse	AGCTGACGACAACCATGCAG	
*Firmicutes* (newly named *Bacillota*)	Forward	ATGTGGTTTAATTCGAAGCA	51
	Reverse	AGCTGACGACAACCATGCAC	
*Prevotella*	Forward	GGTTCTGAGAGGAAGGTCCCC	60
	Reverse	TCCTGCACGCTACTTGGCTG	
*Lactobacillus*	Forward	AGCAGTAGGGAATCTTCCA	58
	Reverse	CACCGCTACACATGGAG	
*Limosilactobacillus reuteri*	Forward	ACCGAGAACACCGCGTTATTT	59
	Reverse	ACCTAAACAATCAAAGATTGTCT	
*Lactobacillus johnsonii*/*gasseri*	Forward	CACTAGACGCATGTCTAGAG	60
	Reverse	AGTCTCTCAACTCGGCTATG	
*Ligilactobacillus murinus*/*animalis*	Forward	TCGAACGAAACTTCTTTATCACC	60
	Reverse	CGTTCGCCACTCAACTCTTT	
*Verrucomicrobia*	Forward	GAATTCTCGGTGTAGCA	59
	Reverse	GGCATTGTAGTACGTGTGCA	
*β-Proteobacteria*	Forward	GGGGAATTTTGGACAATGGG	58
	Reverse	ACGCATTTCACTGCTACACG	
*δ-Proteobacteria*	Forward	GGTGTAGGAGTGAARTCCGT	62
	Reverse	TACGTGTGTAGCCCTRGRC	
*γ-Proteobacteria*	Forward	CMATGCCGCGTGTGTGAA	54
	Reverse	ACTCCCCAGGCGGTCDACTTA	
*Faecalibacterium prausnitzii*	Forward	GATGGCCTCGCGTCCGATTAG	60
	Reverse	GATGGCCTCGCGTCCGATTAG	
*Roseburia*	Forward	GGTGTAAAGGGAGCGCAGG	60
	Reverse	ACTTACCCCTCCGACACTCT	
*Clostridium cocleatum*	Forward	GTAATACATAAGTAACCTGGCCTTT	60
	Reverse	CTCGGATGTCATTTCCTCC	
*Akkermansia muciniphila*	Forward	CAGCACGTGAAGGTGGGGAC	60
	Reverse	CCTTGCGGTTGGCTTCAGAT	

### Statistical analyses

Statistical analyses and graphs were performed with GraphPad Prism 9 software (GraphPad Software Inc., San Diego, CA, USA). Data of body weight, food intake and activity were analyzed with two-way ANOVA (Sex x Time) with Holm–Sidak's or Dunnett’s multiple comparison test. *T* test were used to compared specifically two points. Outliers from qPCR data were identified with Rout 1% statistical test and excluded. qPCR data were then analyzed with two-way ANOVA (ABA x Euthanized time) with Tukey post hoc tests. Data are expressed as mean ± standard error to mean. Concerning all statistical tests, *p* < 0.05 was considered as significant results. In each qPCR graph, significant two-way ANOVA *p* values for the ABA model (ABA), the circadian rhythm (CR) or for interaction (Int) are shown in bold and underlined text (ABA, CR or Int). Tukey post-tests *p* values were mentioned with symbols when *p* < 0.05 and exact *p* values were written when a trend was observed (*p* < 0.1). Exact two-way ANOVA *p* values and the number of animals are also displayed in Additional file 2: Tables S2 and S3, respectively.

## Results

### Body weight, food intake and running wheel activity

**Fig. 1:**
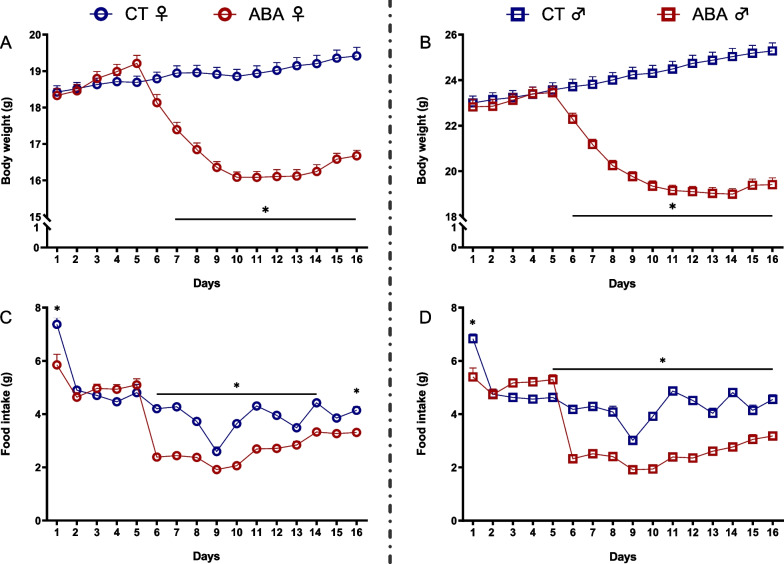
Body weight and food intake. Body weight (**A** and **B**) and food intake (**C** and **D**) measured in female (open circles) and male (open squares) mice submitted to the activity-based anorexia (ABA) model or in control mice (CT). *, *p* < 0.05 vs. CT

As previously reported [1, 50], both female and male ABA mice exhibited a severe body weight loss compared to CT mice (Fig. 1A, andB). Indeed, body weight decreased from day 6 to day 11 and from day 6 to day 14 in female and male ABA mice, respectively. In female mice only, the body weight further increased at days 15 and 16 compared to day 11. On day 16, males and females exhibited 17.2% and 13.2% of body weight loss, respectively, compared to day 5 (before the limited access to food). Body weight loss was associated with a reduced food intake in ABA mice compared to CT mice (Fig. 1C, andD).

**Fig. 2:**
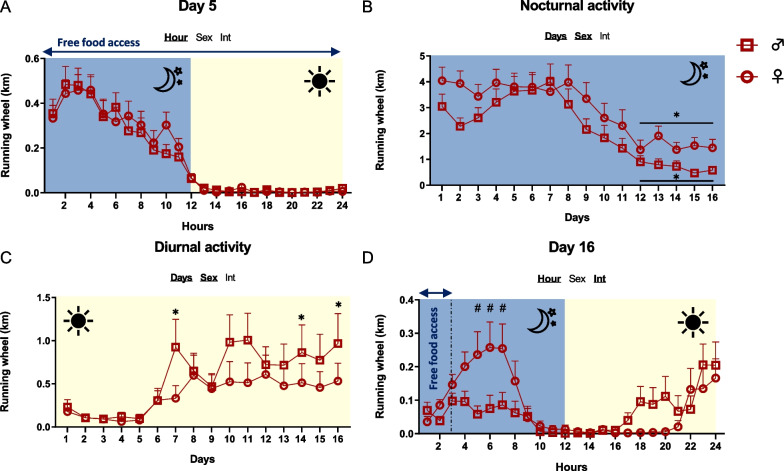
Running wheel activities. Running wheel activities (km) were measured in female (open circles) and male (open squares) mice submitted to the activity-based anorexia (ABA) model. **A** Running wheel activity measured at day 5. **B** Running wheel activity measured during the dark phase from day 1 to day 16. *p < 0.05 vs. day 5. **C** Running wheel activity measured during the light phase from day 1 to day 16. *, *p* < 0.05 vs. day 5. **D** Running wheel activity measured at day 16. #, *p* < 0.05 vs. males. In each graph, significant two-way ANOVA *p* values for the time (Hour), the sex (Sex) or for interaction (Int) are shown by bold and underlined text (*n* = 11–12/group)

During the first 5 days (no limitation of food access), both male and female mice exhibited running wheel activity during the dark phase (Fig. 2B, C). After food access restriction, physical activity during the dark phase remained unchanged until day 7/8 and then decreased in both male and female mice (Fig. 2B). Of note, the level of physical activity during the dark phase remained higher in female than in male mice. By contrast, physical activity during the light phase increased from day 6 in both sexes, although this increase was higher in male mice compared to female mice (Fig. 2C) that is mainly due to the appearance of food-anticipatory activity (Fig. 2D). At the end of the ABA protocol, female mice showed higher wheel activity during the dark phase than male mice (Fig. 2D).

### Central clock genes expression in female and male mice in response to ABA model

**Fig. 3:**
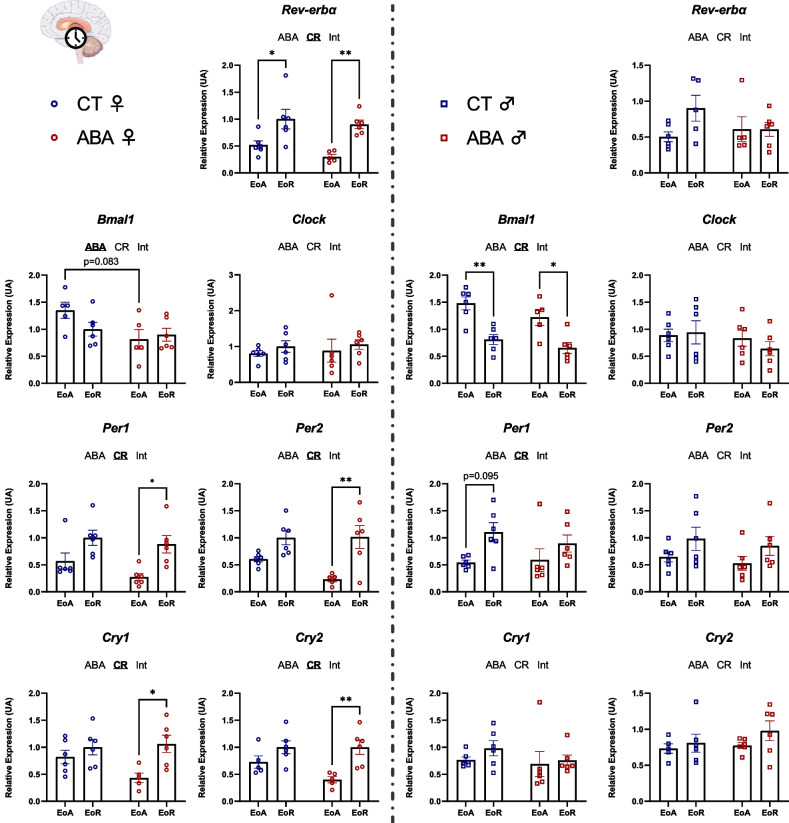
Clock genes expression in the suprachiasmatic nucleus. Clock genes expression (*Rev-erbα, Bmal1, Clock, Per1, Per2, Cry1* and *Cry2*) was quantified in the suprachiasmatic nucleus (SCN) in female (open circles) and male (open squares) mice submitted to the activity-based anorexia (ABA) model or in control mice (CT), at the end of active phase (EoA) or resting phase (EoR). In each graph, significant two-way ANOVA *p* values for the ABA model (ABA), the circadian rhythm (CR) or for interaction (Int) are shown by bold and underlined text (ABA, CR or Int). *, *p* < 0.05 and **, *p* < 0.01 with post-tests (*n* = 5–6 per group)

Expression of clock genes was studied in the hypothalamus area containing the SCN (Fig. 3). In females, *Per1* (*p* < 0.05), *Per2* (*p* < 0.01), *Cry1* (*p* < 0.05) and *Cry2* (*p* < 0.01) mRNA levels were significantly increased at EoR compared to EoA in ABA mice, but not in CT mice (Fig. 3). *Rev-erbα* mRNA expression was similarly modified in CT and ABA mice between EoA and EoR. By contrast, there was no significant changes in *Bmal1* and *Clock* mRNA level according to the circadian phases, even if a trend for a reduction of *Bmal1* mRNA level at EoA was observed in ABA mice compared to CT mice (*p* = 0.083). In male mice, *Bmal1* mRNA expression was significantly different between EoR and EoA (two-way ANOVA *p* value_CR_ < 0.05; Fig. 3) but was not affected by the ABA procedure. Thus, our data underline that only female mice exhibited significant alterations of clock genes expression in the SCN in response to the ABA model.

### Peripheral clock genes expression in female and male mice in response to ABA mode

**Fig. 4:**
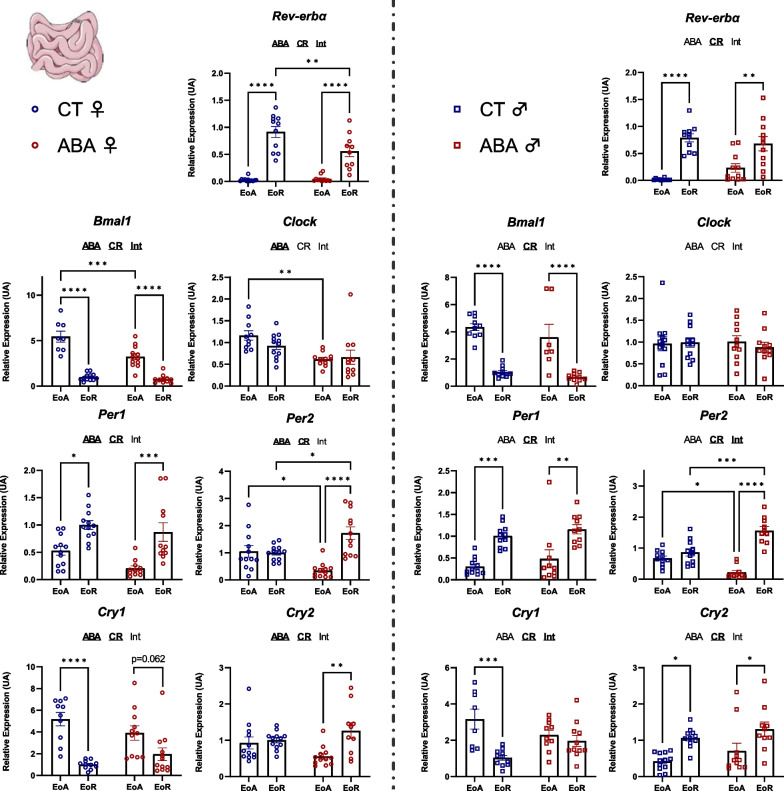
Clock genes expression in the ileum. Clock genes expression (*Rev-erbα, Bmal1, Clock, Per1, Per2, Cry1* and *Cry2*) was quantified in the ileum in female (open circles) and male (open squares) mice submitted to the activity-based anorexia (ABA) model or in control mice (CT), at the end of active phase (EoA) or resting phase (EoR). In each graph, significant two-way ANOVA *p* values for the ABA model (ABA), the circadian rhythm (CR) or for interaction (Int) are shown by bold and underlined text (ABA, CR or Int). *, *p* < 0.05; **, *p* < 0.01; ***, *p* < 0.001 and ****, *p* < 0.0001 with post-tests (*n* = 7–12 per group)

**Fig. 5:**
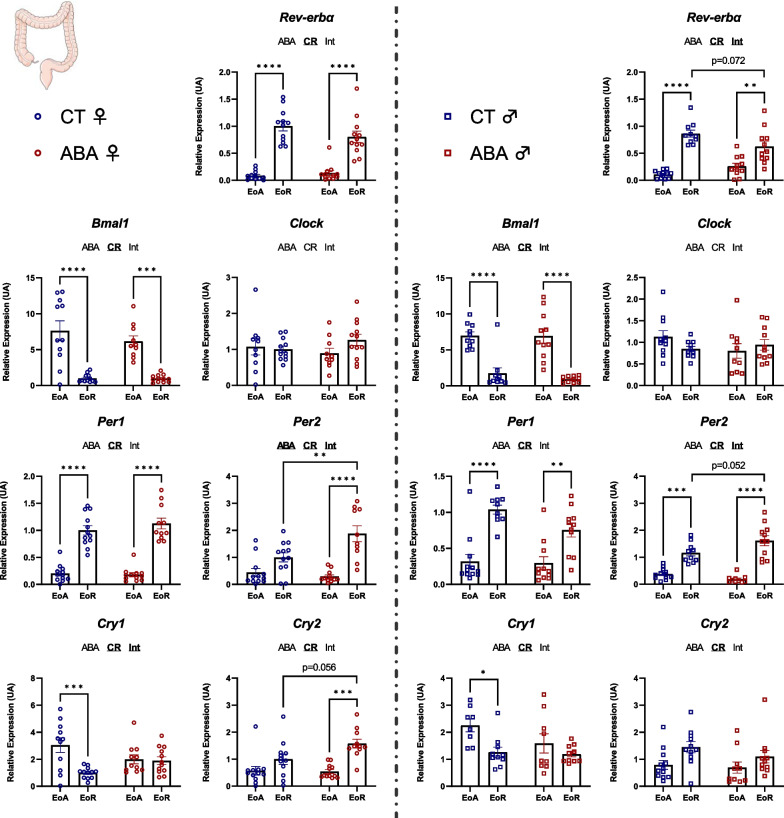
Clock genes expression in the colon. Clock genes expression (*Rev-erbα, Bmal1, Clock, Per1, Per2, Cry1* and *Cry2*) was assessed in the colon in female (open circles) and male (open squares) mice submitted to the activity-based anorexia (ABA) model or in control mice (CT), at the end of active phase (EoA) or resting phase (EoR). In each graph, significant two-way ANOVA *p* values for the ABA model (ABA), the circadian rhythm (CR) or for interaction (Int) are shown by bold and underlined text (ABA, CR or Int). *, *p* < 0.05; **, *p* < 0.01; ***, *p* < 0.001 and ****, *p* < 0.0001 with post-tests (*n* = 8–12 per group)

Then, we evaluated expression of clock genes in peripheral tissues, such as ileum, colon and liver. Interestingly, we observed a sex-dependent response to the ABA model. Indeed, in the ileum, statistical analysis (two-way ANOVA *p* values and post-tests) revealed that all studied factors, namely, *Bmal1, Clock, Per1, Per2, Cry1, Cry2* and *Rev-erbα* showed altered mRNA levels in female ABA mice (for all factors, two-way ANOVA *p* value_ABA_ < 0.05). In males, only *Per2* and *Cry1* mRNA levels were affected by ABA: a circadian variation of Per2 mRNA expression was observed in male ABA mice (*p* < 0.0001) that was not observed in CT (Fig. 4). By contrast, the circadian variation observed for Cry1 in male CT mice (*p* < 0.001) disappeared in ABA mice (Fig. 4). In the colon, ABA induced alterations in *Per2*, *Cry1* and *Cry2* mRNA levels in females. Indeed, female ABA mice exhibited a variation of *Per2* (*p* < 0.0001) and *Cry2* (*p* < 0.001) expression between EoA and EoR, that was not observed in female CT mice (Fig. 5). By contrast, for Cry1, the differences observed in CT mice (*p* < 0.001) was abolished in ABA mice (Fig. 5). Similarly, male CT mice exhibited a significant modification for *Cry1* between EoA and EoR (*p* < 0.05) that was not present in ABA mice. In addition, male ABA mice at EoR exhibited trends for slight modifications for *Per2* (*p* = 0.052) and *Rev-erbα* (*p* = 0.072) expression levels compared to CT mice at EoR. Thus, ABA increased the amplitude of *Cry2* mRNA variation but decreased the amplitude of *Cry1* mRNA variation in both the ileum and colon of female mice. By contrast, in male mice, ABA only decreased the amplitude of *Cry1* mRNA variation.

**Fig. 6:**
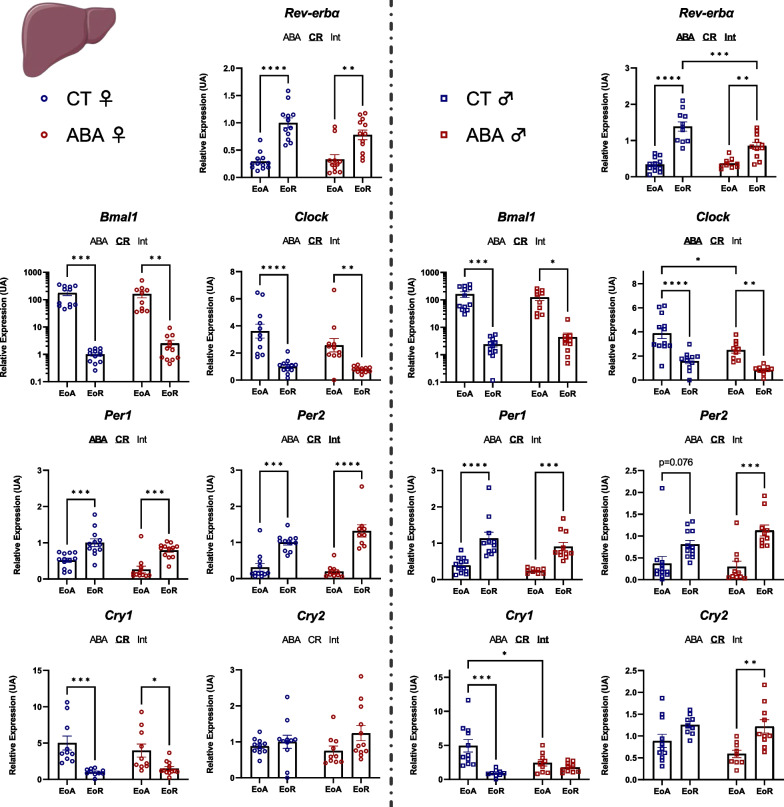
Clock genes expression in the liver. Clock genes expression (*Rev-erbα, Bmal1, Clock, Per1, Per2, Cry1* and *Cry2*) was quantified in the liver in female (open circles) and male (open squares) mice submitted to the activity-based anorexia (ABA) model or in control mice (CT), at the end of active phase (EoA) or resting phase (EoR). In each graph, significant two-way ANOVA *p* values for the ABA model (ABA), the circadian rhythm (CR) or for interaction (Int) are shown by bold and underlined text (ABA, CR or Int). *, *p* < 0.05; **, *p* < 0.01; ***, *p* < 0.001 and ****, *p* < 0.0001 with post-tests (*n* = 9–12 per group)

In the liver, while all studied clock genes showed no changes in response to ABA model in females, except a modest effect for *Per1* (two-way ANOVA *p* value_ABA_ < 0.05), ABA procedure affected *Clock*, *Per2*, *Cry1*, *Cry2* and *Rev-erbα* mRNA expression in males (Fig. 6). Indeed, male ABA mice exhibited significant circadian mRNA levels modification for *Per2* (*p* < 0.001) and *Cry2* (*p* < 0.01) that was not present in CT mice. By contrast, the difference observed in CT mice for *Cry1* (*p* < 0.001) disappeared in ABA mice. Finally, ABA mice exhibited significant differences for *Clock* (at EoA, *p* < 0.05) and *Rev-erbα* (at EoR, *p* < 0.001) compared to CT mice (Fig. 6).

### Interestingly, in all peripheral studied tissues, ABA increased the amplitude of *Per2* in both males and females

To resume, the ABA model per se induced sex- and tissue-dependent alterations of circadian clock genes expression, e.g., major effects on liver for males and on ileum for females.

### Analysis of gut–microbiota according to circadian rhythm in female and male ABA mice

**Fig. 7:**
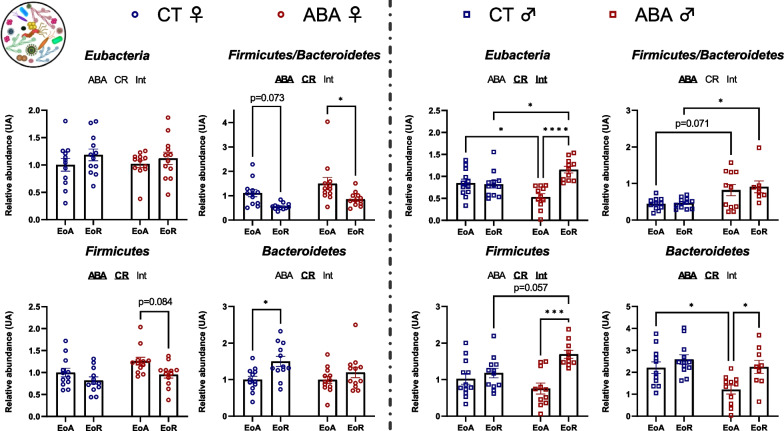
Relative abundance of *Eubacteria*, *Firmicutes* and *Bacteroidetes* in the cecal content. Relative abundance of *Eubateria*, *Firmicutes* and *Bacteroidetes* was quantified in the cecal content of female (open circles) and male (open squares) mice submitted to activity-based anorexia (ABA) model or in control mice (CT), at the end of active phase (EoA) or resting phase (EoR). In each graph, significant two-way ANOVA *p* values for the ABA model (ABA), the circadian rhythm (CR) or for interaction (Int) are shown by bold and underlined text (ABA, CR or Int). *, *p* < 0.05; ***, *p* < 0.001 and ****, *p* < 0.0001 with post-tests (*n* = 8–12 per group)

**Fig. 8:**
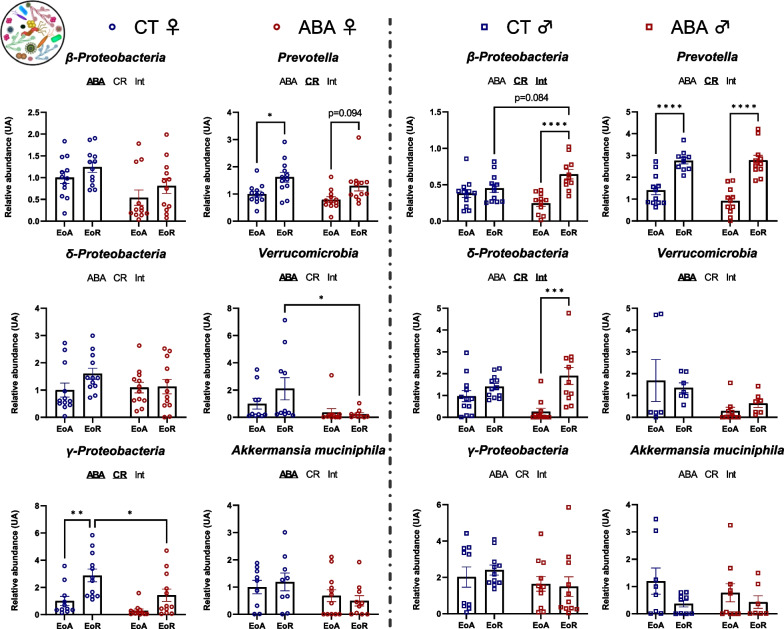
Relative abundance of *Gram negative bacteria* in the cecal content. Relative abundance of *β-Proteobacteria*, *δ-Proteobacteria*, *γ-Proteobacteria*, *Prevotella*, *Verrucomicrobia* and *Akkermansia mucinipila* was quantified in the cecal content of female (open circles) and male (open squares) mice submitted to the activity-based anorexia (ABA) model or in control mice (CT), at the end of active phase (EoA) or resting phase (EoR). In each graph, significant two-way ANOVA *p* values for the ABA model (ABA), the circadian rhythm (CR) or for interaction (Int) are shown by bold and underlined text (ABA, CR or Int). *, *p* < 0.05; ***, *p* < 0.001 and ****, *p* < 0.0001 with post-tests (*n* = 7–12 per group)

**Fig. 9:**
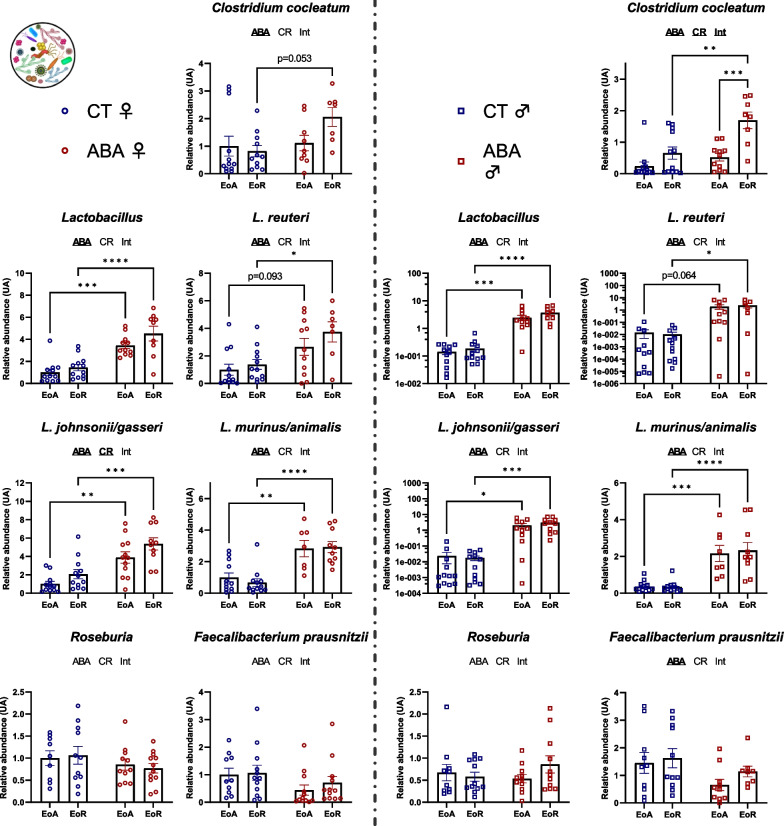
Relative abundance of *Gram positive bacteria* in the cecal content. Relative abundance of *Clostridium cocleatum*, total *Lactobacillus*, *L. reuteri*, *L. johnsonii/gasseri*, *L. murinus/animalis*, *Roseburia* and *Faecalibacterium prausnitzii* was quantified in the cecal content of female (open circles) and male (open squares) mice submitted to the activity-based anorexia (ABA) model or in control mice (CT), at the end of active phase (EoA) or resting phase (EoR). In each graph, significant two-way ANOVA *p* values—for the ABA model (ABA), the circadian rhythm (CR) or for interaction (Int) are shown by bold and underlined text (ABA, CR or Int). *, *p* < 0.05; ***, *p* < 0.001 and ****, *p* < 0.0001 with post-tests (*n* = 7–12 per group)

We then studied gut–microbiota composition by focusing on bacterial *phyla* and families that we had previously reported as changed during the ABA model [7]. We first focused on specific bacterial *phyla* and observed a sex-dependent response (Fig. 7). Indeed, in female mice, *Eubacteria* abundance remained unaffected in response to the circadian rhythm and the ABA model. By contrast, in males, *Eubacteria* abundance varied between EoA and EoR but only in response to ABA (*p* < 0.0001). The *Firmicutes/Bacteroidetes ratio* changed in response to the circadian rhythm in females (two-way ANOVA, *p* value_CR_ = 0.0004), while it was not affected in males. In males, *Firmicutes* and *Bacteroidetes* abundance changed during the circadian rhythm (two-way ANOVA *p* value_CR_ < 0.05) and the ABA model amplified these variations to reach significant differences between ABA–EoA and ABA–EoR (*p* < 0.001 and *p* < 0.05 for *Firmicutes* and *Bacteroidetes,* respectively). Again, for Gram-negative bacteria, we noticed a sex-dependent response (Fig. 8). We then focused on variations in bacterial family abundances. In female mice, we observed a circadian variation of *Prevotella* and *γ-proteobacteria* abundance in CT (*p* < 0.05 and *p* < 0.001, respectively) that did not reach significance in response to ABA model (Fig. 8). By contrast, in males, while *β-proteobacteria* and *δ-proteobacteria* did not show circadian variation in CT mice, ABA mice exhibited a significant circadian variation of these bacteria (*p* < 0.0001 and *p* < 0.001, respectively; Fig. 8). Concerning the Gram-positive bacteria, the ABA model per se induced an increase in *Lactobacillus* in both female and male mice, particularly *L. reuteri*, *L. murinus/animalis* and *L. johnsoni/gasseri* (two-way ANOVA, *p* value_ABA_ < 0.05). We did not observe a circadian variation of *Lactobacillus*, except for *L. johnsoni/gasseri* in females (two-way ANOVA *p* value_CR_ = 0.0204; Fig. 9). Thus, the increase in *Lactobacillus* observed in response to ABA is both sex and circadian rhythm independent (Fig. 9). By contrast, in response to ABA, *Clostridium cocleatum* showed a circadian variation in males (*p* < 0.001, Fig. 9) that was not observed in CT mice.

## Discussion

**Fig. 10:**
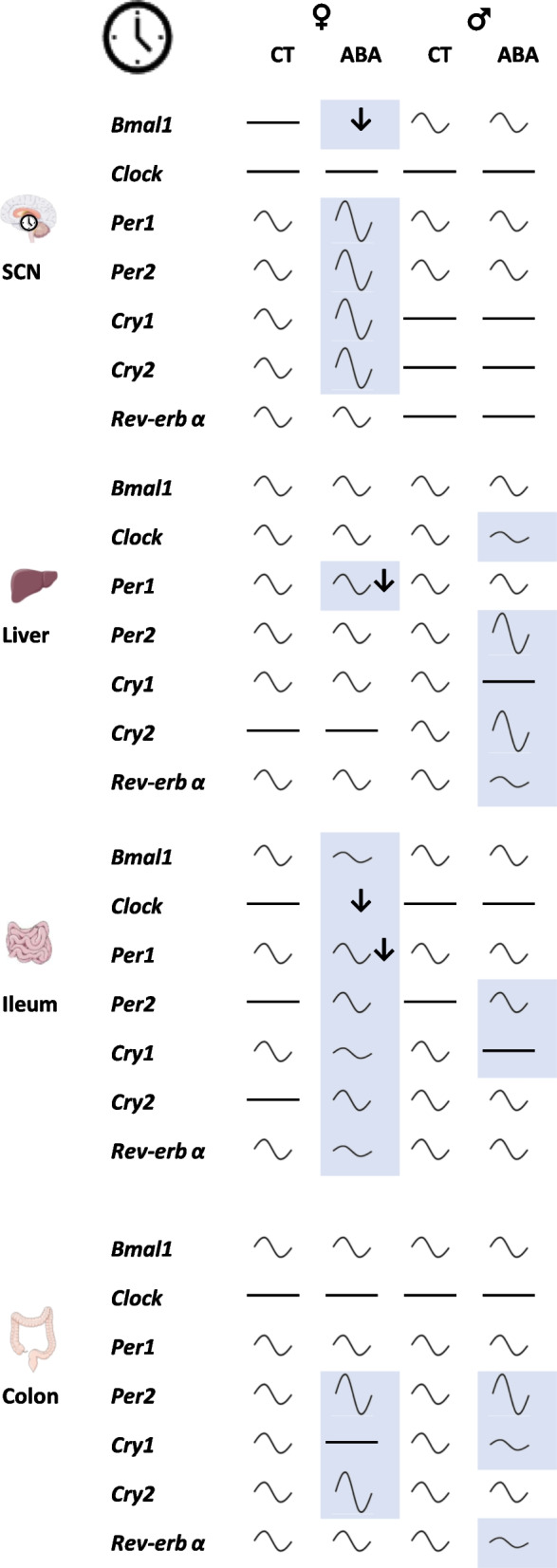
Summary of the effects of the activity-based anorexia (ABA) model on female and male mice clock genes expression in both suprachiasmatic nucleus (SCN) and peripheral tissues. The effects of ABA are highlighted in blue according to the statistical analysis (two-way ANOVA with Tukey post-tests, see statistical methods). Symbols represent: no circadian variation (  ), circadian variation with low (  ), moderate (  ) or large amplitude (  ), reduced (↓) or increased expression (↑)

**Fig. 11:**
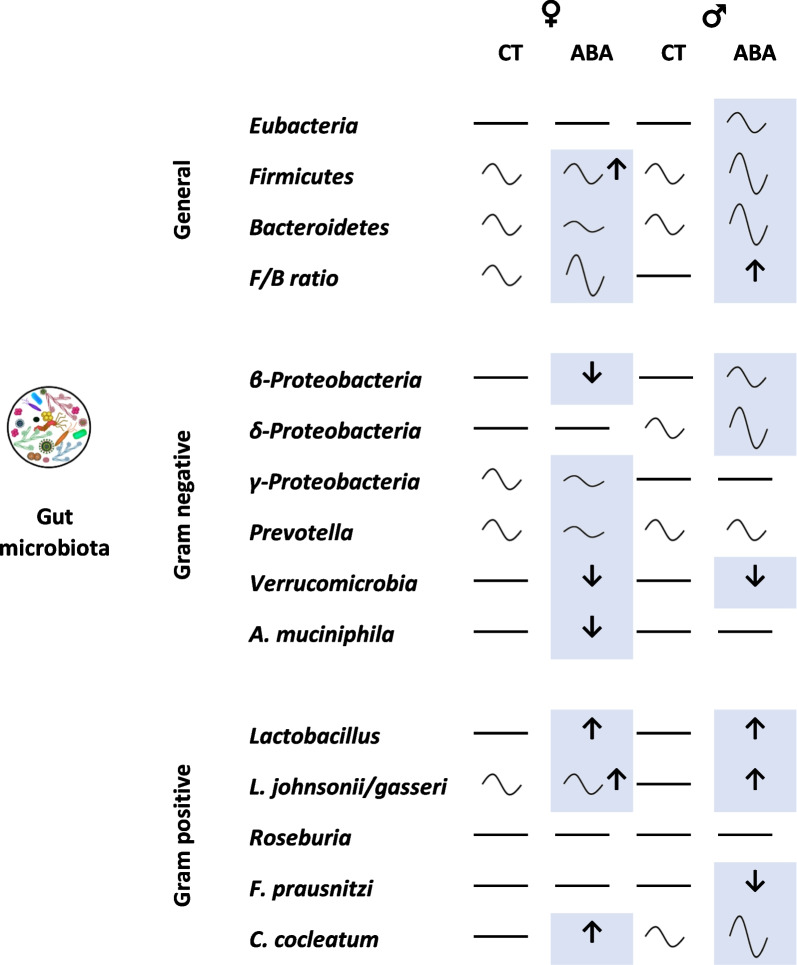
Summary of the effects of the activity-based anorexia (ABA) model on female and male mice gut–microbiota composition. The effects of ABA are highlighted in blue according to the statistical analysis (two-way ANOVA with Tukey post-tests, see statistical methods). Symbols represent: no circadian variation (  ), circadian variation with low (  ), moderate (  ) or large amplitude (  ), reduced (↓) or increased expression (↑)

In the present study, we report for the first time alterations in clock genes expression at both central and peripheral levels in response to the activity-based anorexia (ABA) model (Fig. 10), which is considered to be the most relevant animal model to study the pathophysiology of anorexia nervosa (AN) [43, 44]. In addition, we also show that ABA mice exhibit circadian variations of gut–microbiota composition (Fig. 11). Finally, we emphasize that all these factors are modulated in a sex-dependent manner.

Patients with AN frequently show sleep disorders [2, 3, 31] and circadian hormonal dysregulations [23, 26] that may contribute to the AN pathophysiology, which remains not completely understood. During the last decade, the role of the microbiota–gut–brain axis has emerged in the regulation of feeding behavior [15, 41]. In the ABA model, previous studies reported altered nycthemeral changes with the appearance of physical activity during the light phase [1, 29]. Interestingly this diurnal activity, that mainly corresponds to FAA, is more marked in males compared to females [1]. Li et al*.* have underlined the role of gonadal hormones, since FAA differences induced by restriction feeding were abolished in gonadectomized animals [33]. In the present study, we also observed increased diurnal physical activity that was more marked in male mice. However, until now, no study evaluated the regulation of clock genes expression in mice submitted to the ABA model.

As described in the clock gene database, CircaDB, we observed that clock genes have a specific tissue expression [39]. We focused on the SCN, the master clock mainly controlled by light/dark cycle, and on peripheral tissues: liver, ileum and colon. Interestingly, ABA induced an alteration of clock genes expression in the SCN only in females. Particularly, our data suggest an increase of *Per1, Per2, Cry1* and *Cry2* variations between the end of resting and active phases. In males, we did not observe these changes in response to ABA. Previous studies showed sex-dependent regulation of the clock genes expression in the SCN in response to stress or corticoids [11, 36], while both ABA and AN patients showed activation of the corticotrope axis [4]. Whether these alterations of clock genes expression in the SCN contribute to the higher prevalence of AN in women would deserve further investigations.

In peripheral tissues, ABA mice also exhibited modifications of clock genes expression in a sex-dependent manner. Indeed, in response to ABA model, clock genes expression was more markedly affected in female mice in the ileum, by contrast to the liver, where changes are more pronounced in males. Interestingly, Heddes et al. reported that a disruption of the intestinal circadian rhythm by *Bmal1* intestinal knock-out induced changes in gut–microbiota composition and metabolome [25]. It is now well-established that a gut–microbiota dysbiosis occurs in both AN patients and ABA mice with, in both cases, an increase in *Lactobacillus* levels [5, 7, 20]. In the present study, we confirm the gut dysbiosis in ABA mice compared to CT but we also highlight circadian variations of gut–bacteria species that are amplified in response to ABA model, particularly in males. Even if the gut dysbiosis observed in male ABA mice and female rats may mainly be due to reduced food intake [7, 52], the putative role of intestinal clock genes disturbances on the gut–microbiota needs to be further explored.

Gut–microbiota may affect clock genes expression in both intestinal and extra-intestinal cells [54]. Indeed, germ-free mice exhibited altered intestinal clock genes expression [53] and short chain fatty acids, which are byproducts of bacterial fiber fermentation, were shown to regulate *Bmal1* and *Per2* in hepatocytes [32]. In this study, clock genes expression in the liver was more pronounced in ABA males that also exhibited greater alterations in gut–microbiota circadian variations compared to females.

Clock genes are finally known to regulate several pathways, such as TLR4 expression and inflammatory responses. For instance, a circadian regulation of TLR4 expression has been described in the colon and the SCN [39]. TLR4 circadian expression is regulated by *Bmal1* in macrophages [38] or by RORα and REV–ERBα in intestinal epithelial cells [35]. Further experiments should decipher the role of intestinal clock genes dysregulation on intestinal TLR4 expression, since we previously reported that TLR4 pathway is affected [4] and contributes to the microbiota–gut–brain axis in response to the ABA model [50].

The present study presents several limitations. We used C57BL/6 mice that are deficient in melatonin [42], an important effector of the circadian clock. However, C57BL/6 mice are commonly used for studies on circadian rhythm [19, 28, 39] and have been reported as good responders to ABA [40]. In a recent study in C57BL/6 mice, melatonin injections improved ethanol-induced liver injury by reprogramming the circadian protein BMAL1 [56]. It could be of interest to further evaluate the impact of melatonin on ABA model in C57BL/6 mice. In the present study, to limit the number of animals, we have chosen to limit our evaluation at two timepoints and to compare the circadian clock gene expression in ABA mice to control mice (free access to food and no activity wheel). Further research should also include additional timepoints to obtain a complete kinetic profile. It could be also of interest to compare the data to other groups, such as mice with restricted access to food and no activity wheel or mice with free access to food and to an activity wheel, to evaluate the role of starvation and physical activity, respectively. The impact of food on circadian rhythm has been reviewed [8], as well as the impact of physical activity in humans [46].

### Perspective and significance

To conclude, we report here for the first time a sex-dependent alteration of clock genes expression in the ABA model in both central and peripheral tissues, as well as a modulation of circadian fluctuations in gut–microbiota composition. Our data suggest that an altered circadian rhythm may contribute to the dysfunction of the microbiota–gut–brain axis observed in patients with AN. Further experiments should decipher the underlying mechanisms leading to altered central and intestinal clock genes expression, as well as gut dysbiosis, in anorectic conditions.

### Supplementary Information

**Additional file 1: Figure S1.** Experimental design.

**Additional file 2: Table S1.** Analysable n (n) and retained n after outliers statistical test (ROUT 1%). **Table S2.** Exact two-way ANOVA *p* values (ABA X Euthanized time) of clocks genes expression and gut–microbiota abundances. **Table S3.** Exact Tukey's multiple comparisons test *p* values of clocks genes expression and gut–microbiota abundances.

## Data Availability

The data sets generated and/or analyzed during the current study are available from the corresponding author on reasonable request.

## Abbreviations

ABA: Activity-based anorexia
AN: Anorexia nervosa
Bmal1: Basic helix–loop–helix ARNT-like 1
Clock: Clock circadian regulator
CR: Circadian rhythm
Cry1: Cryptochrome circadian regulator 1
Cry2: Cryptochrome circadian regulator 2
CT: Control mice
EoA: End of activity phase
EoR: End of resting phase
FAA: Food anticipatory activity
Gapdh: Glyceraldehyde-3-phosphate dehydrogenase
Int: Interaction effect of two-way ANOVA
Per1: Period circadian regulator 1
Per2: Period circadian regulator 2
Rev-erbα (newly named Nr1d1): Nuclear receptor subfamily 1 group D member 1
SCN: Suprachiasmatic nucleus
