# Supplementary material for: Sex-dependent circadian alterations of both central and peripheral clock genes expression and gut–microbiota composition during activity-based anorexia in mice
Source: Biol Sex Differ. 2024 Jan 12;15:6. doi: 10.1186/s13293-023-00576-x (PMC10785476; doi:10.1186/s13293-023-00576-x)
Supplement: Supplementary file 1 — Additional file 1: Figure S1. Experimental design. [file 13293_2023_576_MOESM1_ESM.pdf]

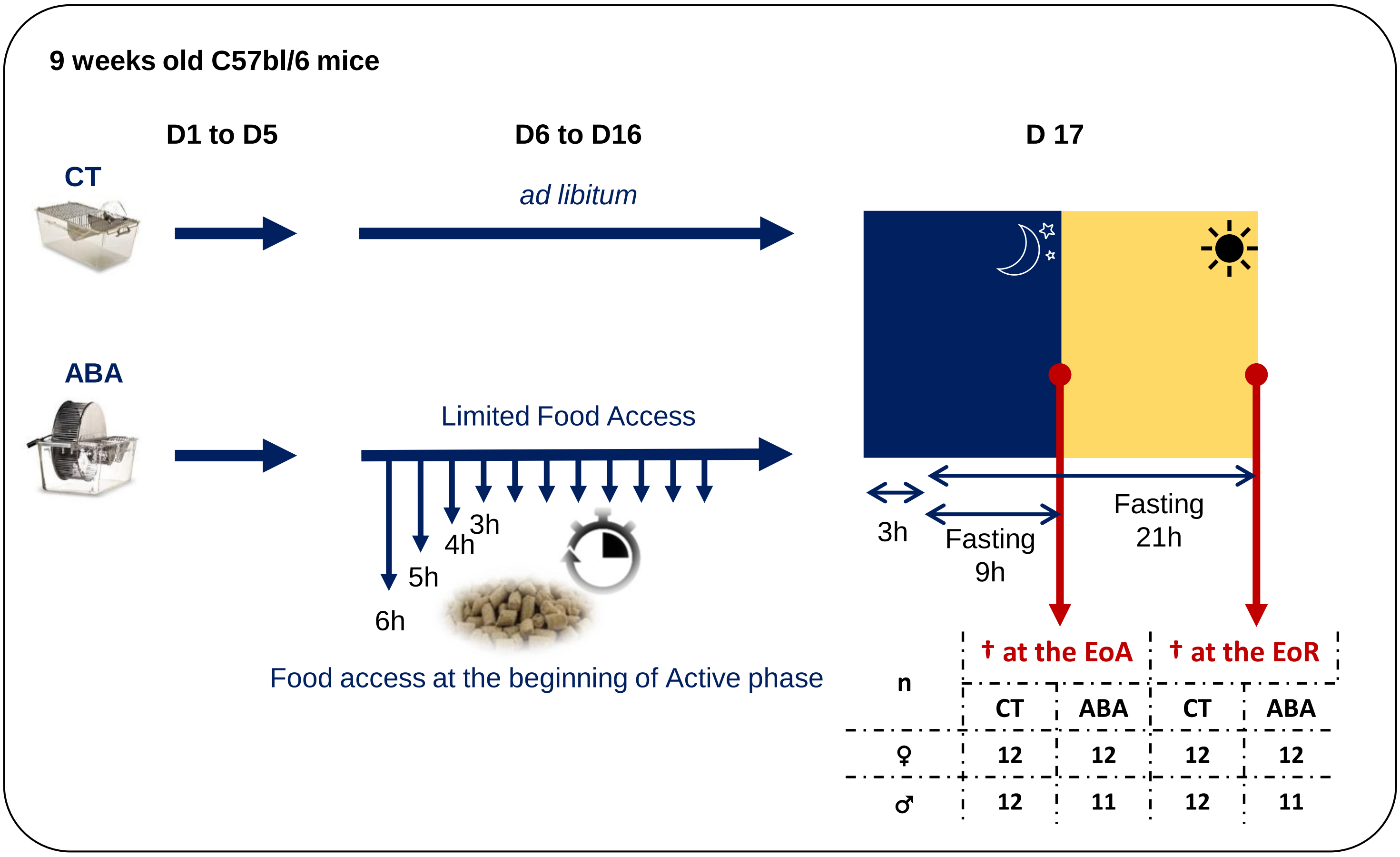

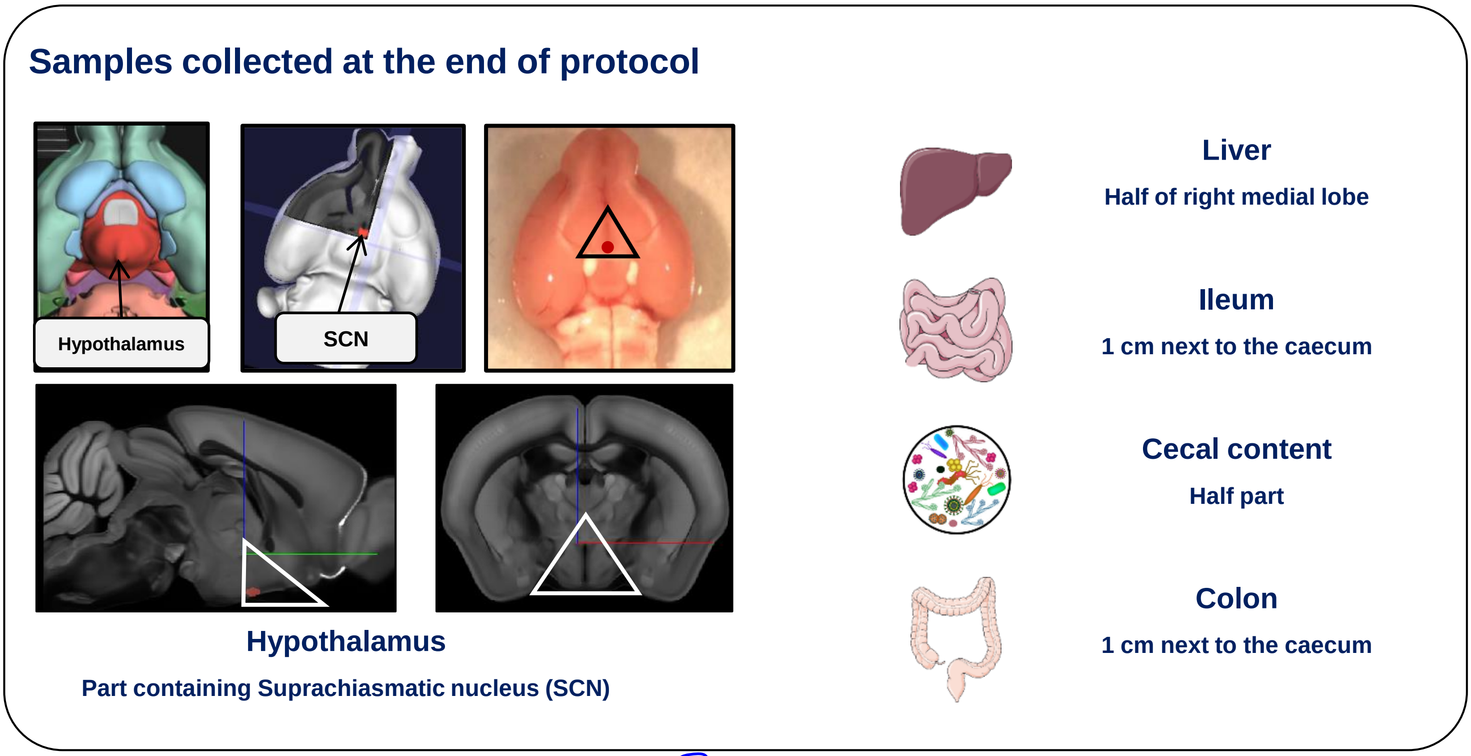

Figure S1: Experimental deisgn

In the upper panel, 9 weeks old male and female C57Bl/6 mice were ramdomized into control (CT) or activity-based anorexia (ABA) model. CT mide had ad libitum access to food. ABA mice had a time-limited access to food and a free access to a running wheel. At the end of the protocol, CT and ABA mice were sacrificed at the end of the resting period (light phase - EoR) or at the end of active period (dark phase - EoA).
In the lower, the samples collected are described.
